# Supplementary material for: Body mass index and physical frailty among older Mexican Americans: Findings from an 18-year follow up
Source: PLoS One. 2022 Sep 9;17(9):e0274290. doi: 10.1371/journal.pone.0274290 (PMC9462817; doi:10.1371/journal.pone.0274290)
Supplement: Attachment — Submitted filename: Response-to-reviewer-comments-BMI-Frailty-07-08-2022.docx [file pone.0274290.s001.docx]

July 08, 2022

**Response to Reviewers' comments**

Reviewer's Responses to Questions

Comments to the Author

**1. Is the manuscript technically sound, and do the data support the conclusions?**

The manuscript must describe a technically sound piece of scientific research with data that supports the conclusions. Experiments must have been conducted rigorously, with appropriate controls, replication, and sample sizes. The conclusions must be drawn appropriately based on the data presented.

Reviewer #1: Yes

Reviewer #2: Yes

Thank you.

________________________________________

2. Has the statistical analysis been performed appropriately and rigorously?

Reviewer #1: Yes

Reviewer #2: Yes

Thank you.

________________________________________

3. Have the authors made all data underlying the findings in their manuscript fully available?

The PLOS Data policy requires authors to make all data underlying the findings described in their manuscript fully available without restriction, with rare exception (please refer to the Data Availability Statement in the manuscript PDF file). The data should be provided as part of the manuscript or its supporting information, or deposited to a public repository. For example, in addition to summary statistics, the data points behind means, medians and variance measures should be available. If there are restrictions on publicly sharing data—e.g. participant privacy or use of data from a third party—those must be specified.

Reviewer #1: Yes

Reviewer #2: Yes

Thank you.

________________________________________

4. Is the manuscript presented in an intelligible fashion and written in standard English?

PLOS ONE does not copyedit accepted manuscripts, so the language in submitted articles must be clear, correct, and unambiguous. Any typographical or grammatical errors should be corrected at revision, so please note any specific errors here.

Reviewer #1: Yes

Reviewer #2: Yes

Thank you.

________________________________________

5. **Review Comments to the Author**

Please use the space provided to explain your answers to the questions above. You may also include additional comments for the author, including concerns about dual publication, research ethics, or publication ethics. (Please upload your review as an attachment if it exceeds 20,000 characters)

**Reviewer #1:** The Study evaluate the relationship between BMI and frailty among non-frail older Mexican Americans in a Longitudinal population-based study of 1,648 non-institutionalized Mexican Americans aged ≥ 67 years from the Hispanic Established Population for the Epidemiologic Study of the Elderly (1995/96-2012/13). Frailty phenotype was defined as meeting three or more of the following: unintentional weight loss of >10 pounds, weakness, self-reported exhaustion, low physical activity, and slow walking speed.

Participants with underweight or obesity category II/ morbid obesity had greater odds of frailty over time compared to those with normal weight after controlling for all covariates. Participants with BMIs in the overweight or category I obesity were at lower odds of frailty over time.

I found the study of interest conducted on a large cohort data. Data support conclusion. Figures and table are exhaustive. The statistical analysis is correct.

**Response:** Thank you!

**Reviewer #2:** The authors examined the relationship between BMI and frailty among non-frail older Mexican Americans at baseline over 18 years of follow up in longitudinal population-based study of 1,648 non-institutionalized Mexican Americans aged ≥ 67 years from the Hispanic Established Population for the Epidemiologic Study of the Elderly (1995/96-2012/13). Frailty phenotype was defined as meeting three or more of the following: unintentional weight loss of >10 pounds, weakness, self-reported exhaustion, low physical activity, and slow walking speed. BMI (kg/m 2) was classified as underweight (<18.5), normal weight (18.5-<25), overweight (25-< 30), obesity category I (30-<35), and obesity category II/morbid obesity (≥35). Covariates included socio-demographics, comorbidities, cognitive function, and depressive symptoms. Generalized Estimating Equation models were performed to estimate the odds ratio (OR) and 95% confidence interval (CI) of frailty as a function of BMI category. Participants with underweight or obesity category II/ morbid obesity had greater odds of frailty over time compared to those with normal weight (OR 2.39, 95% CI 1.29-4.44 and OR 1.62, 95% CI 1.07-2.44, respectively) after controlling for all covariates. Participants with BMIs in the overweight or category I obesity were at lower odds of frailty over time.

**Comment:** The manuscript is interesting, but I have some concerns that should be addressed. Firstly, frailty is actually considered as a “multidimensional” condition. The authors consider only the “physical” domain of the frailty. Please see and discuss “Abete P et al. The Italian version of the "frailty index" based on deficits in health: a validation study. Aging Clin Exp Res. 2017;29:913-926”. I suggest to add in the title “physical” before frailty.

**Response:** We agree with the reviewer comment. We have now added in the discussion under the study limitation section the following “Fifth, our measure of frailty phenotype does not consider cognitive function or psychosocial measures (52)” on page 15, lines 297-298.

**Comment:** Secondly, you have to consider the presence of “sarcopenic obesity” in your patients. Please see and discuss “Remelli F et al. Prevalence of obesity and diabetes in older people with sarcopenia defined according to EWGSOP2 and FNHI criteria. Aging Clin Exp Res. 2022 Jan;34(1):113-120.

**Response:** We have added the following sentence in the discussion section “Another explanation is related to the decreased muscle mass seen in older adults with obesity, known as “sarcopenia obesity”(48, 49)” on page 14, lines 287-289.
