# Supplementary material for: Body mass index and physical frailty among older Mexican Americans: Findings from an 18-year follow up
Source: PLoS One. 2022 Sep 9;17(9):e0274290. doi: 10.1371/journal.pone.0274290 (PMC9462817; doi:10.1371/journal.pone.0274290)
Supplement: Attachment — Submitted filename: Response-to-reviewer-comments-BMI-Frailty-08-22-2022.docx [file pone.0274290.s002.docx]

August 22, 2022

**Response to Reviewers' comments**

**Reviewer #2:**

**Comment # 1:** The modification of title: "I suggest to add in the title “physical” before frailty".

**Response:** The title now reads “Body mass index and physical frailty among older Mexican Americans: Findings from an 18-year follow up”

**Comment # 2:** The references:

- #49: Remelli F et al. Prevalence of obesity and diabetes in older people with sarcopenia defined according to EWGSOP2 and FNHI criteria. Aging Clin Exp Res. 2022 Jan;34(1):113-120.

- #50: Abete P et al. The Italian version of the "frailty index" based on deficits in health: a validation study. Aging Clin Exp Res. 2017;29:913-926”

**Response:** We have added the references. Reference **number 48** correspond to “Remelli F et al. Prevalence of obesity and diabetes in older people with sarcopenia defined according to EWGSOP2 and FNHI criteria. Aging Clin Exp Res. 2022 Jan;34(1):113-120; and reference **number 51** correspond to “Abete P et al. The Italian version of the "frailty index" based on deficits in health: a validation study. Aging Clin Exp Res. 2017;29:913-926”.

Thank you for noticing the absence of the references.

________________________________________
